# Role of Intra-articular Platelet Rich Plasma in the Management of Osteoarthritis: A Review

**DOI:** 10.7759/cureus.3359

**Published:** 2018-09-25

**Authors:** Ujala Zubair, Osama Salam, Zarafshan Zubair

**Affiliations:** 1 Internal Medicine, Dow University of Health Sciences (DUHS), Karachi, PAK

**Keywords:** osteoarthritis, platelet-rich plasma therapy, intra-articular injection

## Abstract

Intra-articular injections are a minimally invasive option developed for the management of patients with joint degenerative conditions. These injections can involve the use of steroid preparations, hyaluronic acid, and blood products. Platelet-rich plasma (PRP) is a cost-effective management modality developed for patients with joint degenerative conditions and has provided promising outcomes. It provides nourishment to the chondrocytes through a rich supply of growth factors and cytokines. This article demonstrates the beneficial effects of PRP therapy in patients with osteoarthritis.

## Introduction and background

Osteoarthritis is defined as a pathological condition involving the cartilage as well as synovium. It is among the top ten causes of disability throughout the globe. It involves progressive damage to the cartilage along with the formation of osteophytes. These changes take place in the presence of inflammation. Previously, osteoarthritis was considered as a condition involving only the cartilage but now this is accepted as a condition involving the whole joint including the synovium, subchondral bone, ligaments, and menisci. It can manifest as synovitis, degenerated ligaments and menisci, and bone remodeling [1].

The management of osteoarthritis can be divided into pharmacologic as well as surgical intervention. Because of the absence of sufficient neuronal and vascular supply, the regenerating ability of cartilage has its limits. Due to limited healing ability, cartilage disorders are a challenging management. The pharmacologic intervention included pain management by using analgesics such as paracetamol, opioids, and non-steroidal anti-inflammatory drugs. Surgical intervention includes microfractures and osteochondral grafts. Another minimally invasive procedure developed for patients with cartilage damage includes intra-articular injections [2].

Intra-articular injections involve steroid preparations, hyaluronic acid, and blood products. It is seen that intra-articular steroid preparations provide symptomatic relief from pain and disability, but these effects are short-lived [3-4]. Corticosteroids disrupt the ongoing inflammatory changes by acting on the nuclear steroid receptors [4-5]. Some of the Federal Drug Administration-approved (FDA-approved) steroid preparations given as intra-articular injections include methylprednisolone acetate, dexamethasone, triamcinolone acetate, triamcinolone hexacetonide, and betamethasone acetate. All of these preparations have similar levels of efficacy and potency [6]. Similarly, the effects of intra-articular hyaluronic acid preparations are also short-lived [7]. The FDA-approved preparations of hyaluronic acid include low and high molecular weight hyaluronic acid and sodium hyaluronate [6]. Hyaluronic acid, a glycosaminoglycan, is naturally present in synovial fluid and acts as a joint lubricant and shock absorber. When given intra-articularly, it acts as an anti-inflammatory agent and analgesic. It provides an improvement in joint function, pain, and quality of life. However, its cost-effectiveness limits its use [8-9]. Platelet-rich plasma (PRP) is a cost-effective management modality developed for patients with cartilage damage.

There are three different methods for preparing platelet-rich plasma; the double spinning method, the single spinning method, and selective blood filtration. A four-eight fold increase in platelet concentration can be achieved by the double spinning method whereas the single spinning method yields one-three fold increase in platelet concentration [10].

Platelets are regarded as the main mediators of hemostasis. They contain alpha granules which are enriched with growth factors. Platelets are also enriched with anti-bacterial and fungicidal agents which provoke the synthesis of interleukins and chemokines. When platelets are activated, this causes the release of growth factors. Among them include growth factors from the transforming growth factor beta family (TGF-beta), platelet-derived growth factor (PDGF), insulin-like growth factor (IGF), and fibroblast growth factor (FGF), etc. In the presence of calcium chloride, the platelet concentrate is activated, which causes the release of these growth factors eventually promoting healing [11]. TGF-beta has many functions in terms of healing. It increases expression of chondrocyte phenotype and causes differentiation of chondrogenic mesenchymal stem cell. It also potentiates the deposition of cellular matrix and counter-interacts many inflammatory mediators, which destroys the cartilage architecture [12-15]. PDGF also has similar functions. PDGF also functions as a chemotactic agent for mesenchymal cells which potentiates healing [16].

PRP has also been found to have anti-inflammatory actions. The inflammatory cascade generated by members of cyclooxygenase family can be inhibited by anti-inflammatory mediators present in PRP [17]. Human growth factor (HGF) in PRP has been found to cause inactivation of NF-kB (nuclear factor kappa-light-chain-enhancer of activated B-cells) transactivation activity [18]. The cannabinoid receptors (CB) present on chondrocytes are involved in anti-inflammatory and analgesic actions. When studied, it was found on exposure to PRP that there is an increase in mRNA (messenger RNA) levels of CB1 and CB2 receptors [19]. IGF-1, present in PRP concentrate can inhibit the apoptosis cascade. Apoptosis is the target of therapy for osteoarthritis. If the degenerating chondrocytes are slowed from progressing towards apoptosis, the overall disease progression is slowed. The expression of various cannabinoid receptors is downregulated by the expression of IGF-1 [20]. To confirm this, an in-vivo study was performed which demonstrated the low levels of apoptosis among chondrocytes in the presence of PRP. Therefore, it was concluded that growth factors present in PRP can only slow down apoptosis in the presence of their interaction with not only chondrocytes but also other joint structures such as the synovium, meniscal cells, bone marrow cells, and fat cells [21].

PRP has an influence on all structures of joint. Chemotactic assays have revealed that the PRP stimulated the differentiation of type-II collagen cells and production of prostaglandins along with the migration of corticospongious bone cells [22].

## Review

Intra-articular preparations involving corticosteroids, hyaluronic acid, and platelet-rich plasma are safe to administer. Adverse effects of corticosteroids are very rare and are usually evident within 6-12 hours of administration. These flares usually resolve within a few days. Rat models have demonstrated that repeated intra-articular corticosteroid administration can lead to cartilage destruction, however, clinical studies have not yet demonstrated cases of cartilage destruction following repeated corticosteroid administration  [23-24]. Hyaloronic acid is naturally present in the joint tissues, hence, it has very few adverse reactions except for some minor local reactions evident in 2%-4% of patients [25-26].

Some authors have compared the use of an intra-articular hyaluronic acid with platelet-rich plasma or platelet-rich growth factors (PRGF). Better pain control was demonstrated among the 30 patients that were given PRGF as compared to the other 30 who received intra-articular hyaluronic acid [27]. In another study, the effectiveness of autologous conditioned serum was compared with hyaluronic acid. Autologous conditioned plasma (ACP) is platelet-rich plasma with low concentration. When both management modalities were compared, it was observed that better pain control and improvement in symptoms was achieved with the use of intra-articular ACP. This study was performed by Creza et al., among 120 patients. Creza et al. concluded that improvement in symptoms was evident in patients with grade-3 knee osteoarthritis [28]. 

Three intra-articular injections of platelet-rich plasma were given to patients with different knee degenerative conditions having a low degree of degenerative changes. These patients were followed up for a 12-month interval. Improved quality of life and knee function was recorded [29]. Another study compared the effects of one PRP and one hyaluronic acid injection with the effect of multiple PRP injections. Patients were followed for six months after the injection. Patients with early osteoarthritis demonstrated beneficial outcomes with multiple PRP injections; however, patients with advanced osteoarthritis showed no benefit with either therapy [30]. 

There are eight meta-analyses conducted on the effectiveness of intra-articular PRP administration. Two of them suggest its beneficial use [7,31]. Four suggest a small benefit associated with its use [32-35] whereas two of them have suggested that PRP therapy has overall no clinical benefit [25-26]. Rutjes et al. report that the results of their meta-analysis, which involved 12,667 patients from 89 clinical trials. They report that there was no clinical improvement on therapy with intra-articular PRP [36].

Some authors have suggested that PRP may have a pro-inflammatory effect, which can worsen the underlying joint damage. It has been observed that the levels of matrix metalloproteinases (MMPs) such as MMP-1 and MMP-2 were increased in synoviocytes suffering from osteoarthritis after incubation with PRP [37]. Others have recommended that PRP initially has a pro-inflammatory response which involves the release of pro-inflammatory cytokines. However, this initial response is followed by an anti-inflammatory response. This response manifests as an inhibition of the release of interleukins, cyclooxygenases, and metalloproteinases [38].

Few studies have compared the rates of proliferation with different concentrations of platelet stimulate. Gaissemaner et al. in their study results reported that cellular proliferation was evident at platelet stimulating concentration up to 10% whereas no proliferative activity was demonstrated at concentrations above 10% [39]. Similarly, Yang et al. concluded that the minimum concentration required to stimulate the proliferation of chondrocytes was 1%. At concentrations above 10%, mass formation can be evident [40]. Spreafico et al. found that 5% was the optimal concentration of platelet release (PR) required to stimulate chondrocyte proliferation among concentrations of 1%, 5%, and 10% [41]. Intra-articular PRP injections increase the amount of type-II collagen and prostaglandins. Platelet-poor plasma (PPP), as well as fetal bovine serum (FBS), can also raise the amount of type-II collagen and prostaglandins; however, they cannot raise their concentration to the extent PRP does [42]. 

## Conclusions

PRP therapy can have beneficial effects, but the final outcome depends on factors such as age, gender, BMI, and degree of degenerative changes. Multiple PRP injections can provide a benefit to patients with a low BMI and a lesser degree of degenerative changes. Further research on the pro-inflammatory role of PRPs is needed.
